# The multisensory body revealed through its cast shadows

**DOI:** 10.3389/fpsyg.2015.00666

**Published:** 2015-05-19

**Authors:** Francesco Pavani, Giovanni Galfano

**Affiliations:** ^1^Center for Mind/Brain Sciences, University of Trento, Rovereto, Italy; ^2^Department of Psychology and Cognitive Science, University of Trento, Rovereto, Italy; ^3^Department of Developmental and Social Psychology, University of Padua, Padua, Italy; ^4^Center for Cognitive Neuroscience, University of Padua, Padua, Italy

**Keywords:** shadow, spatial attention, multisensory, body perception, self-recognition, touch, vision

## Abstract

One key issue when conceiving the body as a multisensory object is how the cognitive system integrates visible instances of the self and other bodies with one’s own somatosensory processing, to achieve self-recognition and body ownership. Recent research has strongly suggested that shadows cast by our own body have a special status for cognitive processing, directing attention to the body in a fast and highly specific manner. The aim of the present article is to review the most recent scientific contributions addressing how body shadows affect both sensory/perceptual and attentional processes. The review examines three main points: (1) body shadows as a special window to investigate the construction of multisensory body perception; (2) experimental paradigms and related findings; (3) open questions and future trajectories. The reviewed literature suggests that shadows cast by one’s own body promote binding between personal and extrapersonal space and elicit automatic orienting of attention toward the body-part casting the shadow. Future research should address whether the effects exerted by body shadows are similar to those observed when observers are exposed to other visual instances of their body. The results will further clarify the processes underlying the merging of vision and somatosensation when creating body representations.

## Introduction

The processing of shadows has been the target of an increasing number of studies in recent years. The results stemming from this line of investigation have demonstrated that information conveyed by shadows can support several tasks performed in everyday life. It is now well established that our visual system can process shadows very rapidly (e.g., Elder et al., 2004; Rensink and Cavanagh, 2004) and use shadows for several visual functions (see Mamassian et al., 1998; Dee and Santos, 2011; for reviews). For instance, it has been shown that shadows can foster object recognition (Norman et al., 2000; Mascalzoni et al., 2009). Moreover, several studies have shown that cast shadows of objects can play a critical role in defining the spatial arrangement of objects within a scene, in both dynamic and static contexts (e.g., Kersten et al., 1997; Yonas and Granrud, 2006; Imura and Tomonaga, 2009). Furthermore, reaching movement kinematics can also be affected by the shadow casted by the target object (Bonfiglioli et al., 2004).

One very special class of objects casting shadows in the environment is represented by human bodies. Others that we perceive in the visual scene often cast shadows of their body or body parts. Moreover, our own body is frequently a source of shadows, projecting images of our bodily self in the environment. It is now widely acknowledged that full bodies or body parts represent special stimuli for the brain and they are processed by specialized neural pathways (e.g., Downing et al., 2001; Arzy et al., 2006; Pourtois et al., 2007; Calvo-Merino et al., 2010; Cazzato et al., 2015). This special salience of body-related stimuli is also well reflected in behavioral effects, which suggest that body parts undergo prioritized processing compared to other objects (e.g., Ro et al., 2007; Igarashi et al., 2008, 2010), especially when they belong to one’s own body (e.g., Frassinetti et al., 2009; Ferri et al., 2011).

Recently, researchers have asked whether shadows cast by body parts may represent a unique class of stimuli for the visuomotor system. More specifically, the focus of research has covered two related, yet distinct issues, i.e., the generic effects of someone else’s body shadow vs. the specific effects of one’s own body shadows on cognitive processing. One first relevant question in this literature is whether shadows cast by bodies can also undergo prioritized processing compared to other objects—similar to what has been documented for visible instances of bodies. A second important question is whether body shadows may trigger reflexive orienting of attention toward the body that casts them. It has been proposed that, when seeing a cast shadow, our visual system is somehow forced to find an association between the visible shadow and the object that most likely casts it, thus solving the so-called “shadow correspondence problem” (Mamassian, 2004). While for generic objects this could serve the main purpose of reducing the perceptual complexity of the visual scene by promoting perceptual bindings between segmented elements, in the case of body shadow it could serve a different yet fundamental function: deciding which visible instances of bodies in the scene belong to the self and which belong to others. When applied to body shadows, the shadow-correspondence problem may thus be central to a perceptual decision that ultimately promotes self-identification and self-recognition.

The primary aim of the present review is to provide a comprehensive perspective of the studies that examined how cast shadows of bodies affect our cognitive processes. We will first discuss the limited literature on the influence on behavior of shadows cast by the body of others, and then we will turn to the issue of shadows cast by our own body. This organization has been adopted with the goal of introducing the more specific topic of the review (the influence of one’s own shadows when creating body representations) starting from a more general perspective. In particular, we will examine (1) how cast shadows of our own body can change a sense of bodily space, by promoting binding between personal space and the space occupied by one’s own shadow; (2) whether cast body shadow of our own body can “push” attention toward the body itself; (3) the extent to which this orienting effect may occur automatically. We will conclude by discussing the implications of this literature for the study of body perception in general and outlining some possible development of this research field, which is still in its infancy.

## The Effects of Someone Else’s Body Shadows

Research in this subtopic has primarily converged on the attempt to address the basic question of whether someone else’s body shadow can affect one’s motor behavior. Tentative evidence supporting a positive answer was provided by Liden and Herberholz (2008), who investigated whether fake shadows resembling the body of a predator might influence movement in crayfishes. To this purpose, they used an experimental setting in which an object moving at different velocities effectively mimicked the shadow of an attacking predator. Crayfishes exhibited two different types of escape responses whose prevalence critically depended of the velocity of the moving shadow.

As concerns humans, Alaerts et al. (2009) conducted a study in which participants were required to watch video clips in which either a hand of a stranger or its cast shadow were shown executing abduction/adduction movements of the index finger while transcranial magnetic stimulation was administered over the hand-related area of the primary motor cortex and electromyographical activity was recorded from the muscle of the participants’ index finger. Motor-evoked potentials showed an increased amplitude for both the real hand and the hand shadow conditions as compared to when movements were performed by an unrecognizable object (control condition). This pattern of results has been taken to support the idea that visible body parts and body shadows alike are sufficient to activate motor areas, as long as a biological movement is implied. In a similar study which combined electromyography and transcranial magnetic stimulation, Sartori and Castiello (2013) addressed the mirror neuron system’s ability (for a review, see Rizzolatti and Craighero, 2004) to resonate with movements shown in full illumination vs. shadowed movements, in which the hand performing a reach-and-grasp sequence was shown with the little finger in shadow. Note that in this study the manipulation involved attached rather than cast shadows (i.e., shadows falling on the body, rather than the shadow projected by the body). Motor-evoked potentials for shadowed movements exhibited a decrease in amplitude as compared to the full illumination condition. Sartori and Castiello (2013) interpreted this finding as suggesting that body shadow processing can be reflected at the level of the human mirror neuron system, even when shadows are not relevant for the task at hand.

Turning to behavioral studies, recent evidence has been reported indicating that observing a cast shadow of one hand can affect imitative behaviors in humans. Badets et al. (2013) presented their participants with two superimposed visual stimuli (one on the foreground and the other on the background). One of the two stimuli depicted a hand and the other depicted its cast shadow. The participants were required to imitate the movement (opening vs. closing the fingers) of one stimulus (the target) while ignoring the other (the distractor). Crucially, there were congruent trials (in which the hand and the shadow performed the same movement) and incongruent trials (in which the hand and the shadow executed opposite movements). In addition, there was a real shadow condition (in which the shadow always appeared on the background and the hand appeared in the foreground), and a no-shadow condition (in which the shadow appeared in the foreground and the hand appeared on the background, i.e., a situation which is known to break one of the shadow priors, see, e.g., Casati, 2003). A response time distributional analysis demonstrated that participants suffered from an interference effect (i.e., they were slower in initiating movements on incongruent trials as compared to congruent trials). Crucially, this effect vanished for the slowest responses in the real shadow condition only. Badets et al. (2013) have argued that imitation abilities can be deeply influenced by body shadows. They interpreted the fact that interference was present (also for slowest responses) in the no-shadow condition as suggesting that participants likely treated these stimuli as real hands.

Recently, the role of body shadows cast by others has been investigated also in the context of computer vision and robotics (Dee and Santos, 2011). A particularly interesting applied research domain in this regard is related to person identification for vision-based surveillance systems. Aerial search and surveillance systems typically rely on a top view of the human body, with much less details than in side views. Iwashita et al. (2012) have demonstrated that shadows provide additional information regarding body biometrics that enhance person identification and gait recognition both inside a building (using artificial light) and outside (under the natural sunlight). It would be interesting to extend this line of research to animal species that use aerial view (e.g., birds), to explore to what extent cast shadow can also constitute a cue for object recognition. Furthermore, although humans typically do not see other humans from an aerial perspective, it would be interesting to examine to what extent adding shadow stimuli could promote recognition of people in natural scenes (e.g., Reeder and Peelen, 2013).

## One’s Own Body Shadows Bind Personal and Extrapersonal Space

The data reviewed in the previous section indicate that body shadows (of others) can have a strong impact on the visuomotor system, in both humans and other animal species. One’s own body shadows, however, may be even more salient. Each shadow cast by our own body broadly refers to a location (the body part casting it) for which we have exteroceptive, proprioceptive and interoceptive experience. This feature makes body shadows potentially capable to contribute to the construction of the internal representation of body shape and its extension in space.

A pivotal role in starting this line of investigation has been played by the work of Pavani and Castiello (2004). In their experiments, Pavani and Castiello used a very popular experimental setting in multisensory research, that is the visuo-tactile interference paradigm (e.g., Pavani et al., 2000; Spence et al., 2004a,b). The participants performed a tactile elevation discrimination task (with thumb and index finger arranged one below the other, judge which of the two fingers was stimulated) while ignoring a simultaneous task-irrelevant visual stimulus. The typical finding observed with this setting is that tactile localization performance is worse when tactile and visual stimuli occur at different elevations (e.g., touch at the index, vision at the thumb) compared to when they occur at the same elevation (e.g., touch and vision both at the index finger). Crucially, this visuo-tactile interference is greater when the visual distractors are presented near the stimulated hand, compared to when they are presented further away from the body (Spence et al., 2004a).

Interestingly, Pavani and Castiello (2004) observed that task-irrelevant visual stimuli presented far and equidistant from both hands but in close proximity to the shadow cast by one of the two hands produced a much stronger interference effect when tactile targets were delivered to the hand casting the shadow as compared to when they were presented at the other hand. Such modulation was genuinely related to body shadows, as it vanished when participants wore a shaped glove projecting an unnatural polygonal shadow or viewed a line drawing silhouette of a hand. Pavani and Castiello (2004) argued that participants reacted to the visual stimuli near the shadow of the hand as if the stimuli were affecting the hand itself. Also in consideration of previous reports that visuo-tactile interference can be observed also when visual distractors are presented to fake hands aligned to the real hands (see Pavani et al., 2000) and that it can be influenced by active tool-use (e.g., Maravita et al., 2002a), Pavani and Castiello (2004) have interpreted the magnification of visuo-tactile interference as evidence that body shadows may create some sort of binding between personal and extrapersonal space (i.e., the space occupied by the body and the space occupied by the shadow, respectively).

The notion that our own body shadows can be incorporated into our personal multisensory space of the self (see Cardinali et al., 2009; de Vignemont, 2011; for reviews), has recently been supported also by findings reported by Kuylen et al. (2014), who used a perceptual matching task. Based on the idea that the ability to interact with an object at any distance shrinks the perceived distance between object and observer (e.g., Witt et al., 2005), Kuylen et al. (2014) tested whether viewing the shadow of one’s own body extending toward a target object may result in the subsequent underestimation of the distance between the body and the same target object. The results confirmed that, compared to a baseline condition in which no body shadow was visible, the participants exhibited an estimation bias to report a shorter distance when the body shadow was present. Interestingly, this phenomenon, was also reliable when participants interacted with the target object by means of a tool (a laser pointer), but it did not emerge when the body shadow was replaced by the shadow projected by a different object (a large file cabinet placed behind the participant which covered the shadow cast by the body). This latter finding clearly indicates that cast shadows of our own body are different from other types of shadows and suggests that they may indeed act as extensions of the body, as originally proposed by Pavani and Castiello (2004).

Before exploring the effects of one’s own body shadows for body perception further, it is worth noting that owned body shadows have also been studied in applied cognitive science, especially in the context of user interface research. Devices exploiting shadows cast by the body of users have been implemented for operating graphical information on large displays (e.g., Xu et al., 2006). These shadow-based interfaces enable users to interact with a computer by simply using the shadows cast on the screen by the upper limbs (and more specifically by the fingers). Takeuchi et al. (2014) have demonstrated that body shadows can be very effective as pointing cursors. This may be due to the fact that users do not have particular difficulties in understanding the correspondence between the movement of the fingertips and the movement of the related cast shadow. Specifically, the cognitive ergonomics validity of using one’s own body shadows for the interaction with distal surfaces may relate to the natural tendency of our cognitive system to bind personal and extra-personal spaces through one’s own body shadows.

The research reviewed so far, stemming from different disciplines and perspectives, highlights that body shadows are highly peculiar stimuli. Interestingly, unlike tools or other objects such as rubber hands, they are immaterial and can only provide visual information (they are not multisensory stimuli). Another important point is that, unlike other objects that are capable of shaping the subjective extension of the body in space, the type of visual information they convey is quite coarse, being only two-dimensional. Although the two-dimensional nature of cast shadows does not prevent extracting useful three-dimensional information about the object casting it (Norman et al., 2009), the correspondence between the 2D cast body-shadow and the 3D body part remains underspecified. There cannot be a 1:1 mapping between points on the shadow and points on the body. The shadow of one’s head, for instance, could relate to either the front or the back of the head (we thank one of the reviewers for this interesting remark).

## One’s Own Body Shadows Shift Attention to the Body

**FIGURE 1:**
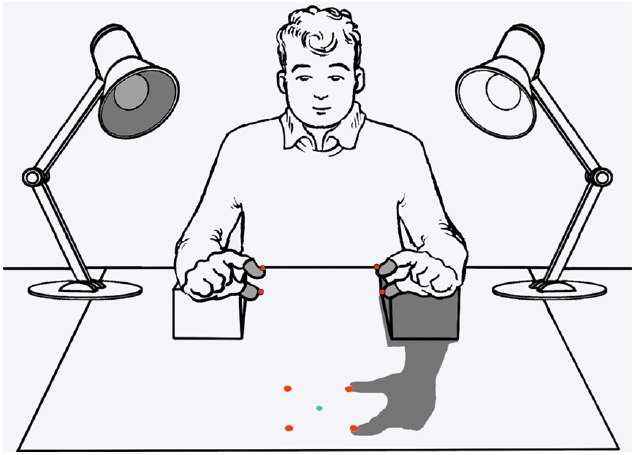
**Schematic illustration of the experimental setting in the experiments investigating orienting of attention mediated by body shadows adapted from Pavani et al. (2014).** A trial with a left-hand shadow is shown. Tactile stimulators are embedded in the gray sheaths around the index and thumb of each hand. Red LEDs were used only in experiments addressing visual modality in personal (at the hand) and extrapersonal (near the shadow cast by the hand) space and were illuminated one at a time. The green LED served as fixation point. See the text for details.

Pavani and Galfano (2007, Galfano and Pavani, 2005; Pavani et al., 2014) have addressed another critical possibility concerning the role of body shadows, namely, the possibility that they can serve as important cues to the multimodal sense of body. Galfano and Pavani (2005) hypothesized that body shadows may indeed represent a high-priority class of stimuli that act by “pushing” attention toward the body itself. To this purpose, they modified the paradigm used by Pavani and Castiello (2004) to implement an exogenous or reflexive spatial cueing paradigm (e.g., Jonides, 1981; see Spence and Santangelo, 2009; for a review in the context of multisensory research), in which hand shadows served as spatially uninformative visual cues (Figure 1). The participants were delivered tactile targets unpredictably to the thumb or index finger of either hands and were asked to localize them irrespective of the stimulated hand. At the same time, they viewed the shadow of either the touched or untouched hand cast in front of them by a lateral light source. In the first experiment, the hand casting the shadow remained fixed within a block of trials, but the participants were explicitly told that the tactile target had the same probability to be delivered on the hand casting shadow and in the other hand. This, in turn, made the shadow entirely irrelevant for the task at hand. Nevertheless, localization performance was better when targets touched the hand casting the shadow (spatially congruent trials) than the other hand (spatially incongruent trials). This pattern was very robust, suggesting that body shadows somehow cued attention back the body part casting it. Although the body shadow conveyed no predictive information about the target location, in a second experiment the hand casting the shadow varied unpredictably from trial to trial. This manipulation had the purpose of discouraging participants from adopting implicit strategies to deliberately attend to the hand casting the shadow. The results, again, showed that tactile localization performance was significantly better at the hand casting shadow than at the other hand. This finding was taken as evidence that the attentional cueing effect toward the body part casting the shadow was indeed genuinely reflexive rather than the consequence of some top-down strategy.

Galfano and Pavani (2005, Experiment 4) also conducted an experiment in which participants were prevented from seeing their own hands. This manipulation had the purpose of ruling out the possibility that the observed spatial cueing effect resulted from the fact that the visible hand casting the shadow was illuminated more strongly than the other hand. Orienting of attention mediated by body shadows was still present, suggesting that the alternative account could be dismissed. For another experimental condition, in which the cast shadow of an object (a piece of cardboard) overlapped and completely masked any shadow cast by the hand, the data showed no reliable effects. This latter pattern rules out yet a further alternative account which would attribute the better performance on spatially congruent trials over spatially incongruent trials to the fact that the lateralized light source that was turned on to create the shadow might also potentially convey somatosensory (thermal) stimulation to the hand casting the shadow. Such account can be rejected because the asymmetrical thermal stimulation (if any) was present also in the object-shadow condition and no differences in performance emerged. The observed pattern clearly demonstrated that shadow-driven orienting was specific to body shadows. However, it is worth noting that the object shadow condition did not differ from the body shadow condition only for the shape of the shadow. Indeed, the object shadow was stationary throughout each block of trials, whereas body shadow was obviously spatio-temporally correlated to the movements, if any, of the hand (likely inducing a sense of agency).

The possible role of the sense of agency (for a review, see Tsakiris et al., 2007) as a key factor for accounting for the orienting of attention mediated by body shadows reported by Galfano and Pavani (2005) has been explored by Pavani and Galfano (2007). They specifically addressed whether shadow-induced benefits on tactile localization performance were dependent on the correspondence between the seen shadow and the object casting it, that is self attribution of the visible image (i.e., shadows) of the body. To this aim, they implemented three different cue conditions in their experimental set up. Beside the standard hand-shadow condition, similar to Pavani and Castiello (2004), Pavani and Galfano (2007) also included a condition in which participants wore a shaped glove casting an unnatural polygonal shadow (real shadow with unnatural shape), and a condition in which participants were presented with photographs consisting of shadow-like images projected from above (fake shadow with natural shape). This allowed dissociation of two different factors that may be at work for endorsing self attribution of shadows and to estimate their impact in isolation. In the real shadow with unnatural shape condition, self attribution, if any, was promoted by spatio-temporal movement correlation between hands and their shadows alone. In contrast, in the fake shadow with natural shape condition, the only factor at work was represented by the visual similarity between the hands and the (static) shadow-like images. Overall, participants exhibited a significantly faster tactile localization performance for cued over uncued hands only for the real shadow condition. In a more in-depth analysis aimed to uncovering possible fluctuations of orienting of attention mediated by body shadows within each block of trials, Pavani and Galfano (2007) observed an interesting pattern of data also for the other experimental conditions. The analysis revealed that for the fake-shadow with natural shape condition, a reliable shadow-mediated orienting effect was present in the first part of each experimental block. In sharp contrast, in the real shadow with unnatural shape condition this effect was significant in the last portions of each block only. The overall findings were taken as strong evidence that orienting of attention mediated by body shadows is critically bound to self attribution of shadows. The temporally diverging trend for the fake-shadow with natural shape condition and the real shadow with unnatural shape condition was interpreted as evidence that the sense of ownership of shadows is strongly mediated by both spatio-temporal correlation between hands and shadows (i.e., a sense of agency) and visual similarity, although these two factors operate in a different fashion (e.g., van den Bos and Jeannerod, 2002; Whiteley et al., 2004; Tsakiris et al., 2005, 2006).

Another critical question addressed by Pavani and Galfano (2007) is whether attention shifts induced by body shadows comprise the whole portion of visual space they occupy or the body part referred to by shadows exclusively. In so doing, they modified the basic paradigm by adding visual targets located at either the external boundaries of the hand shadow (i.e., close to fixation and far from the hand), or at the index finger and thumb of both hands. The results showed that, overall, shadow-mediated orienting was reliable for tactile targets only, strongly suggesting that body shadows push attention to the body part they refer to, rather than cueing the portion of space they cover.

## The Attentional Link between One’s Own Shadows and the Body is Fast and Mandatory

One important question that arises from the studies that showed that one’s own body shadows can orient attention to the body—and specifically to touches on the body—is whether this effect is mandatory. Recently, Pavani et al. (2014) have addressed the automaticity of attention shifts elicited by body shadows by focusing on two different features that are widely assumed to characterize exogenous orienting of spatial attention: the speed of attention orienting and its sensitivity to contextual modulations. It is important to reiterate that in all the experiments reported by both Galfano and Pavani (2005) and Pavani and Galfano (2007), the body shadow effectively cued attention to the body part casting the shadow despite shadow being spatially non-predictive of the target location. While this is considered a critical feature of reflexive orienting (e.g., Galfano et al., 2011, 2012), another feature that is often deemed as a hallmark for automatic processing is that this type of orienting typically results in a very early rising effect (e.g., Müller and Rabbitt, 1989; Cheal et al., 1994). In behavioral studies, this latter feature is reflected in the observation of a significant benefit in performance for spatially congruent over spatially incongruent trials with very short (below 200 ms) cue-target stimulus onset asynchrony (SOA). Because both Galfano and Pavani (2005) and Pavani and Galfano (2007) invariably used a fixed 2750-ms SOA between cue (the cast shadow of the hand) and target (tactile or visual), Pavani et al. (2014) manipulated SOA and included also a 100-ms SOA. This very short SOA is known to reveal reliable spatial orienting effects with other types of attentional cues, such as eye gaze (e.g., Driver et al., 1999; Galfano et al., 2012). The results showed a robust orienting of attention mediated by body shadows early in processing and sustained over time, as it was not modulated as a function of SOA for both tactile targets (Pavani et al., 2014; Experiment 1) and visual targets delivered near the shadow and far from the hands, i.e., in extrapersonal space (Pavani et al., 2014; Experiment 2).

The second feature addressed by Pavani et al. (2014) was whether shadow-driven orienting is resistant to contextual modulations. It is a widely shared assumption that strongly automatic processing should be impervious to changes in the experimental setting and task demands (e.g., Zbrodoff and Logan, 1986; Ristic and Kingstone, 2005; Pavan et al., 2011). Pavani et al. (2014) addressed this criterion of automaticity by intermixing target modality in the same experiment. Unlike previous experiments, in which target modality remained fixed, their participants responded to unpredictable tactile and visual targets. These latter targets were delivered near the shadow and far from the hands (i.e., in extrapersonal space; Pavani et al., 2014, Experiment 3) or directly at the hands (i.e., in personal space; Pavani et al., 2014, Experiment 4). The results showed a reliable orienting of attention mediated by body shadows for tactile targets in agreement with the previous studies in which touch was the only target modality (Galfano and Pavani, 2005; Pavani and Galfano, 2007). However, the effect for targets in the visual modality became inconsistent, irrespective of whether they appeared in personal or extrapersonal space (i.e., near or far from the hand). Overall, these findings provide support for the notion that orienting of attention mediated by body shadows for tactile targets is a strongly automatic phenomenon, as it appears early in processing and is unaffected by contextual changes (e.g., Zbrodoff and Logan, 1986). In sharp contrast, orienting of attention by body shadows was visible for visual targets (in extrapersonal space) only to the extent that sensory modality was fixed. Hence, orienting to visual targets cannot be said to be strongly automatic as it is clearly sensitive to contextual manipulations (also see Pavani and Galfano, 2007).

Taken together, the studies on orienting of attention triggered by our own body shadows indicate that cast shadows of body parts may indeed represent a high-priority class of stimuli. They act by “pushing” attention toward the body itself and this effect has the characteristics of a mandatory process, at least for the tactile modality. Seeing our own body shadow is a powerful cue toward tactile sensations at the body part casting the shadow. The effect is also influenced by self-attribution of the cast shadow: its presence is tightly linked to perceived ownership of the cast shadows. When this attribution fails, cast shadows can quickly become ineffective as a cue for attention. In the next paragraphs, we examine the extent to which the effects observed for body shadows may extend to other types of visual instances of the body in the environment.

## Are Body Shadows Special?

One important issue in relation to the observations reviewed here for body shadows is to the extent to which they imply mechanisms specific to shadows only, or instead constitute examples of more general processes, such as those involved in multisensory body perception. Consider, for instance, the shadow correspondence problem briefly illustrated in the Introduction section. The problem for the cognitive system is to find the correct correspondence between the seen cast shadow and the object in the environment to which it belongs (Mamassian, 2004). The findings reviewed above, showing that vision of task-irrelevant shadows of one’s own body automatically triggers attention orienting to touches on the body, might stem from the solution of the body-shadow correspondence problem. This interpretation would link the observed findings to a process which has been proposed specifically for cast shadows. An alternative possibility, however, is that a somewhat similar process exists also whenever we experience visible body parts in the environment. During our waking life, images of our own body are almost always present and available in first person perspective. Furthermore, we have third-person views of ourselves through mirrors, photos, videos and nowadays also virtual-reality setups and avatars. Because the body of others is also a frequent stimulus in the environment, occasionally in first-person view and most often in third-person view, choosing which of these visual instances of bodies correspond to our own corporeal awareness is a fundamental task that our cognitive system is constantly asked to solve.

By analogy with the shadow correspondence problem, one could argue for a more general “visible-body correspondence problem,” and posit the existence of a cognitive process whose aim is to correctly match the seen bodies with our own corporeal awareness. This process would involve binding instances of the body across sensory modalities (vision and somatosensation) and, sometimes, across different spatial locations (extra-personal and personal)—just like it occurs with body shadows. Solving the visible-body correspondence problem could be at the roots of the discrimination between body images that belong to oneself and body images that belong to others, strengthening self-other distinction, bodily self-recognition and, ultimately, the psychological experience of the self.

Thus, the key question is whether we can generalize from the body-shadow correspondence problem to a more general “visible-body correspondence problem.” If this is the case, it should be possible to find parallels between the results that emerged from the literature on body shadows and the more general literature on multisensory body perception. Specifically, there should be evidence (1) that a seen body part in the environment (i.e., a photograph or video of one’s own hand) “pushes” attention to the corresponding body part; (2) that this process occurs particularly for touch (or somatosensation); and (3) that this process is largely automatic. As we shall see in the next paragraphs, although several studies in the literature do suggest that visible body parts can affect somatosensory processing, parallels between the findings reviewed here for own body shadows and the studies on own pictorial images of the body are still limited.

When searching for effects of seen body parts on tactile perception one key phenomenon described in the literature is the so-called “Visual enhancement of touch” (VET). VET emerges as improved tactile detection and discrimination at a specific body part (typically a hand), when the body part is either seen directly (Kennett et al., 2001; Taylor-Clarke et al., 2002, 2004; Press et al., 2004; Whiteley et al., 2004) or through a pictorial representation (either video or photograph; Tipper et al., 1998, 2001). Critically, VET emerges despite the fact that vision of the body part is completely task-irrelevant and uninformative about somatosensation. This multisensory effect has been reported in neurologically healthy participants, but there is also evidence that vision of body parts can ameliorate the somatosensory deficits in brain-damaged patients (Serino et al., 2007; see also Rorden et al., 1999; for related findings with vision of a rubber hand).

In many VET studies, the importance of self-attribution of the seen hand remains unclear. This is because tactile enhancements were measured as the difference in performance between a condition in which participants observed an owned body part vs. an object. This contrast does not allow to determine whether the crucial factor is seeing “a” hand, or seeing the “owned” hand (for discussion see Longo et al., 2008). There have been two attempts to address this issue. Haggard (2006) asked participants to discriminate the orientation of gratings delivered to the index finger tip, under three different viewing conditions. Participants either viewed their own hand, or viewed a neutral object, or viewed the hand of a third person aligned with the tactually stimulated hand. Compared to the viewing of a neutral object, both viewing one’s own body part and viewing the body part of another person produced orientation discrimination enhancements. This finding seems to suggest that VET can generalize also to the viewing of body parts that belong to others. In a subsequent study, however, VET emerged specifically for the self-attributed visible body parts. Longo et al. (2008) asked participants to perform a similar orientation discrimination task, while viewing a rubber hand that appeared in the felt location of the real hand through a mirror. To manipulate the perceived ownership of the visible rubber hand, they stimulated the real and fake hands in synchrony (leading to an illusion of ownership) or out of synchrony (no illusion of ownership) across blocks (also see Botvinick and Cohen, 1998). The results showed that VET boosted performance particularly for those participants who performed the tactile discrimination task near threshold. Importantly, they also showed that among participants performing near threshold, VET was larger when the rubber hand was self-attributed compared to when it was considered an extraneous body part. Thus, it appears that under certain circumstances modulations of tactile performance in the presence of visible body parts can be strengthened by self-attribution, similar to the case of body shadows.

In the typical VET experiment, the viewing condition is continuous during the entire block of trials. Tipper et al. (2001, Experiment 2) tested VET using an experimental setup that allowed timed presentation of the visible body part and control over the temporal interval between the onset of the viewing condition and the tactile stimulation. They used three cameras to project displays of different body parts of the participant: face, neck or hand. On each experimental trial, one of these visual displays was shown, either 200 or 700 ms before the tactile target. Participants were instructed to detect touches at a specific body part (e.g., the face), while ignoring distractors at another body site (e.g., the neck). The results showed that response speed advantages emerged regardless of the onset asynchronies between the visible body part and the tactile target. A recent EEG study by Cardini et al. (2012) has provided consistent evidence that VET reflects a phasic effect and can be elicited by very brief exposure to one’s body part. Overall, these findings on VET are reminiscent of the early-rising effect of body shadows on tactile targets documented by Pavani et al. (2014). In that study, orienting of attention mediated by body shadows occurred even at the 100 ms SOA, and this effect was particularly stable and robust for tactile targets.

A different, yet related, line of research worth mentioning is the one that explored the interpretation of mirror reflection of body parts. To correctly interpret mirror-reflections, our brain needs to understand that the object that appears in the mirror (e.g., our face) occupies in fact a different location in space. This process is clearly similar to the shadow-correspondence problem, and it is probably the closest match to the cognitive mechanism at play when interpreting shadows in the environment. Furthermore, it is classically considered evidence of self-awareness in human development and ethology (Gallup, 1982). Interestingly, there is evidence that human infants typically succeed in interpreting mirror-reflections of themselves by their 2 year of life (Gallup et al., 2002). As for body shadows, shadow self-recognition appears to emerge at age 3 (Cameron and Gallup, 1988).

Using the visuo-tactile interference paradigm later used also by Pavani and Castiello (2004) for body shadows, Maravita et al. (2002b) explored the interpretation of mirror reflections of body parts. They asked participants to perform a speeded spatial discrimination for touches at the hands, while ignoring concurrent visual distractors. Critically, in one condition the visual distractors were physically close to the participant’s hands, but were seen only as distant mirror reflections; in another condition they were physically in far space, and appeared near a dummy hand or the hand of another person. The results showed that the strongest visuo-tactile interference emerged for the mirror condition, suggesting that participants recoded the true source of the visual distractors near the body. Similar to the body-shadow studies, vision of the hands (mirror reflected, dummy, or someone else’s) was completely task irrelevant. One interpretation of this finding is that participants mandatorily remapped the self-attributed hand to the actual space the hand occupied, hence coding visual distractors close to the mirror-reflection of the hands from far to near space.

## Future Directions for a Novel Research Field

Research on body shadows is still in its infancy. However, it has the potential to provide a window onto the cognitive and neural mechanisms that regulate the multisensory construction of body representation and the bodily-grounded sense of the self. More generally, it can provide useful insights on the multisensory representation of space, on shadow perception in general, or even on the principles that make shadows a useful and ergonomic tool for human-computer interfaces. While these multiple directions are all worth exploring, we suggest here four possible future developments for this new research domain.

The first one, builds on the considerations offered in the previous section, and is concerned with the possibility of exploring the effects of body shadows on somatosensory perception and self-processing further, with the goal of finding parallels between processing of body shadows and processing of other seen instances of the body in the environment. For instance, it would be very interesting to understand the extent to which body shadows and other seen instances of the body could trigger attention to somatosensation in general. At the moment, all the studies conducted on body shadows examined their effects for spatial touch. Whether similar cueing effect could also exist for other aspects of somatosensation, such as pain perception, proprioception or interoception is unknown. Exploring this aspect would help understanding the extent to which seeing body shadows may be a cue for all bodily sensations—i.e., a cue for the body in general. Interestingly, indications that seeing one’s own body, through direct vision or mirrors, can affect somatosensation in general and not just touch, are already available in the literature. For instance, looking at an image of ourselves in the mirror has been shown to improve the perception of heart-beat signals, and specifically heart-beat counts which are considered a proxy of the person’s ability to pay attention to interoceptive signals (Ainley et al., 2012). There is also evidence that vision of one’s own body parts can modulate pain perception (Longo et al., 2009, 2012; Romano et al., 2014). Another line of investigation within this aim of finding parallels between perception of body-shadow and perception of other visible instances of the body, is related to the validation of the existing findings obtained for shadows of body-parts, to shadows of the whole-body. In recent years, seminal works using virtual reality approaches have already took the study of multisensory body perception in this direction using whole-body illusions (e.g., Ehrsson, 2007; Lenggenhager et al., 2007; Slater et al., 2010) and the study of the interactions between whole-body and body-part perception is also very promising (Liang et al., 2015). At present, however, there has been no attempt to explore the effects of whole-body shadows on body perception.

A second direction worth exploring concerns the neural correlates of body shadow perception. A number of studies in the last decade have examined the neural correlates of visible bodies or body-parts (e.g., Pourtois et al., 2007; Cazzato et al., 2015). In addition, studies have documented the influences of visible body parts on somatosensory processing, primarily exploring the neural correlates of the VET effect described above (Macaluso et al., 2000; Taylor-Clarke et al., 2002; Sambo et al., 2009; Gillmeister and Forster, 2010). These studies have revealed that task-irrelevant vision of body parts can modulate somatosensory processing, including the earliest stages involving the primary somatosensory cortex (Longo et al., 2011). It would be informative to unravel whether the same neural mechanisms described for the visible bodies are also recruited during vision of body shadows. Also, given the importance of self-attribution of body shadows in cueing attention to the body (Pavani and Galfano, 2007), it would be interesting to explore the role of possible right-hemispheric specializations for self-processing (Keenan et al., 2000; Sugiura et al., 2005) using a neuropsychological approach. Behavioral evidence has suggested that implicit self-attribution of seen body parts can enhance performance on match-to-sample body discrimination tasks—a phenomenon which has been labeled “self-advantage” (Frassinetti et al., 2008). The use of this paradigm in brain-damaged patients has revealed an interesting dissociation, with left-brain damaged patients retaining self-advantage in body discrimination tasks, whereas right-brain damage patients performing equally regardless of whether the seen body part belong to themselves or not (Frassinetti et al., 2008, 2010; see also Frassinetti et al., 2012; for similar results in brain-damaged children from the age of 4 years). If right-hemispheric lesions undermine implicit self-recognition, then they should also impair the mechanisms of orienting of attention toward the body triggered by self-attributed body-shadows.

A third direction concerns the effects of body shadows of others. As reviewed above, the literature on this topic is currently very limited and it has primarily explored the consequences on motor behavior of participants observing images of others or images of their shadow acting in the environment. As already anticipated, it would be interesting to examine to what extent body shadows of others could promote person recognition in complex natural scene (Reeder and Peelen, 2013). Furthermore, building on the literature on one’s own body shadows, it might be expected that body shadows of others could also trigger attention to the individuals that cast them—a process that could in itself also foster detection of conspecifics in the environment.

Finally, moving from mechanisms of body perception to more general mechanisms of visual processing, it would be interesting to understand whether some of the principles that have emerged from the literature on body-shadow could apply also to processing of shadows cast by non-bodily objects. For instance, there is evidence that shadows can be treated as objects in the scene (albeit at a coarse spatial scale; see Lovell et al., 2009) and as such can favor within-object advantages for attention orienting (de-Wit et al., 2012). It is unknown, however, whether the cast shadow and the object to which it belongs are bound together at some stage of visual processing, into a unique perceptual entity. The literature on body shadows would suggest that this is the case and that cueing the shadow could result in attention being directed to the object, but this is currently an open empirical question.

## Conclusion

In the present review we pursued two aims. First, we attempted to provide the first systematic account of the effects of body shadows on behavior, considering both the studies that examined the effects of body shadows cast by other people and the relatively larger literature on the effects of body shadows cast by one’s own body. The latter literature, in particular, revealed that shadows cast by one’s own body can promote binding between personal and extrapersonal space and can orient attention toward the body-part casting the shadow. These effects emerge despite body shadows being completely task-irrelevant and they conform to several of the features that characterize automatic processes.

The second aim of the present review was to examine to what extent the effects documented for body shadows may be specific to shadows only or may also extend to other multisensory processes involving the body perception and attention. Although we delineated possible parallels between the effects of cast shadows of one’s own body and the effect of viewing other visual instances of one’s own body, it is clear that this remains an open empirical question. We believe that addressing this issue in future studies will be highly informative. If processing of body shadows is somewhat unique, then this would imply the existence of a cognitive and neural mechanism that developed (perhaps through phylogenesis) to quickly resolve and exploit the redundant information provided by cast shadows in the environment. In this scenario, it would be important to assess whether such a process is selective for body shadows or generalizes to the processing of shadows cast by any of the objects in the environment. By contrast, if processing of body shadows is similar to that involved in the processing of other visual instances of the body, then the studies reviewed here could offer insights into the more general mechanisms that subtend the complex but necessary task of merging vision and somatosensation when constructing body representations.

## Author Contributions

All authors provided substantial contributions to the conception or design of the work; or the acquisition, analysis, or interpretation of data for the work; and contributed in drafting the work or revising it critically for important intellectual content; and approved the final version for publication; and agreed to be accountable for all aspects of the work in ensuring that questions related to the accuracy or integrity of any part of the work are appropriately investigated and resolved. The authors are grateful to Paola Rigo and Tommaso Sega for the artwork in Figure 1.

### Conflict of Interest Statement

The authors declare that the research was conducted in the absence of any commercial or financial relationships that could be construed as a potential conflict of interest.
